# Enhancing the stability of CT radiomics across different volume of interest sizes using parametric feature maps: a phantom study

**DOI:** 10.1186/s41747-022-00297-7

**Published:** 2022-09-15

**Authors:** Laura J. Jensen, Damon Kim, Thomas Elgeti, Ingo G. Steffen, Lars-Arne Schaafs, Bernd Hamm, Sebastian N. Nagel

**Affiliations:** Klinik für Radiologie, Charité – Universitätsmedizin Berlin, corporate member of Freie Universität Berlin and Humboldt- Universität zu Berlin, Hindenburgdamm 30, 12203 Berlin, Germany

**Keywords:** Radiomics, Reproducibility of results, Texture analysis, Tomography (x-ray computed)

## Abstract

**Background:**

In radiomics studies, differences in the volume of interest (VOI) are often inevitable and may confound the extracted features. We aimed to correct this confounding effect of VOI variability by applying parametric maps with a fixed voxel size.

**Methods:**

Ten scans of a cup filled with sodium chloride solution were scanned using a multislice computed tomography (CT) unit. Sphere-shaped VOIs with different diameters (4, 8, or 16 mm) were drawn centrally into the phantom. A total of 93 features were extracted conventionally from the original images using PyRadiomics. Using a self-designed and pretested software tool, parametric maps for the same 93 features with a fixed voxel size of 4 mm^3^ were created. To retrieve the feature values from the maps, VOIs were copied from the original images to preserve the position. Differences in feature quantities between the VOI sizes were tested with the Mann-Whitney *U*-test and agreement with overall concordance correlation coefficients (OCCC).

**Results:**

Fifty-five conventionally extracted features were significantly different between the VOI sizes, and none of the features showed excellent agreement in terms of OCCCs. When read from the parametric maps, only 8 features showed significant differences, and 3 features showed an excellent OCCC (≥ 0.85). The OCCCs for 89 features substantially increased using the parametric maps.

**Conclusions:**

This phantom study shows that converting CT images into parametric maps resolves the confounding effect of VOI variability and increases feature reproducibility across VOI sizes.

**Supplementary Information:**

The online version contains supplementary material available at 10.1186/s41747-022-00297-7.

## Key points

- Parametric maps provide a method to increase the reproducibility of radiomic features.
- The confounding effect of the variability of volume of interest is reduced by using a fixed voxel size.
- Visualising the features in parametric maps can provide insights into their behaviour.

## Background

Since 2012, radiological images have been analysed with radiomics. The underlying rationale is that clinical images contain quantitative information, reflecting the underlying pathophysiology of the examined tissue [1, 2]. The image substructures are analysed mathematically, resulting in quantifiable features with different levels of complexity [3]. The standard approach is to correlate the feature quantity to clinical endpoints such as tumour phenotypes, treatment response, or survival [4–7]. Although numerous publications suggest different features or radiomics signatures as helpful decision-making tools, radiomics are not applied in clinical routine until today [8].

The lack of reproducibility is considered a major drawback of radiomics. All steps around feature extraction may influence their quantity: image acquisition and reconstruction parameters, segmentation, and applied software [3, 9–18]. Noise is furthermore presumed to fundamentally affect radiomic features derived from computed tomography (CT) images [19]. The image biomarker standardisation initiative, IBSI, an international collaboration, attempts to standardise radiomic feature calculation concerning definition and nomenclature [20]. Still, they did not provide guidelines for feature calculation settings [21].

Recent studies emphasised that the findings of radiomic studies may also be caused or influenced by differences in the volume-of-interest (VOI) size. For example, Traverso et al. [22] investigated 841 CT-derived radiomic features from head and neck and lung cancers and identified a correlation with the tumour volume in almost 30% of the features. Another CT study concerning radiation-induced lung disease found 11 of 27 textural features strongly influenced by volume sizes in simulated tumour volumes in the contralateral, nonaffected lung parenchyma [23]. And also, the developers of PyRadiomics, a software tool to extract radiomic features, already state that the size of the segmented volume confounds different first-order features due to the underlying mathematical calculations [24].

In 2021, Kim et al. introduced their tool for creating parametric maps [25]. The basic principle is to calculate maps for the whole image by dividing it into voxels with a fixed size. This way, all features are calculated for VOIs (*i.e.,* each single voxel of the parametric map) of the same size. The results are stored in parametric maps with the same spatial information as the original image, and feature values can be directly recovered the same way one would measure Hounsfield units in any standard image viewer. On the contrary, when performing a conventional extraction, features are calculated for the entire segmented volume, where the size of the underlying VOI can vary.

We, therefore, aimed to explore an approach to correct the confounding effect of VOI variability of CT-derived radiomics by preprocessing the images into parametric maps before feature extraction. The stability of the radiomic features across different VOI sizes was compared between the conventional radiomic feature extraction from the original CT images and the feature extraction from the parametric maps.

## Methods

### Phantom and CT scanning details

The concept of the water phantom was already published in 2021 [26]. We used a plastic cup filled with 100 mL sodium chloride as the imaging phantom. Its homogenous structure ensured that all measured effects were evoked by the altering VOI size and not alterations of the inner texture. CT images were acquired on a 320-detector row CT scanner (Aquilion ONE, Canon Medical Systems, Otawara, Japan) using the small field of view. The phantom was scanned ten times to prevent effects by outliers. To simulate conditions of repeated examinations with slightly varying positioning, the phantom was placed in the isocentre, removed after each scan, and repositioned for the subsequent acquisition. Scan parameters are shown in Table 1.

**Table 1:** CT scanning details

Parameter	
Tube voltage (kVp)	120
X-ray tube current (mA)	50
Exposure time (s)	0.5
Single collimation width	0.5
Total collimation width	100
Reconstruction kernel	Body
Slice thickness (mm)	0.5
Pixel spacing (mm)	0.430/0.430
Matrix	512 × 512
Field of view (mm)	220 × 220

*kVp* Peak kilovoltage, *mA* Milliampere

### Conventional feature extraction

**Fig. 1:**
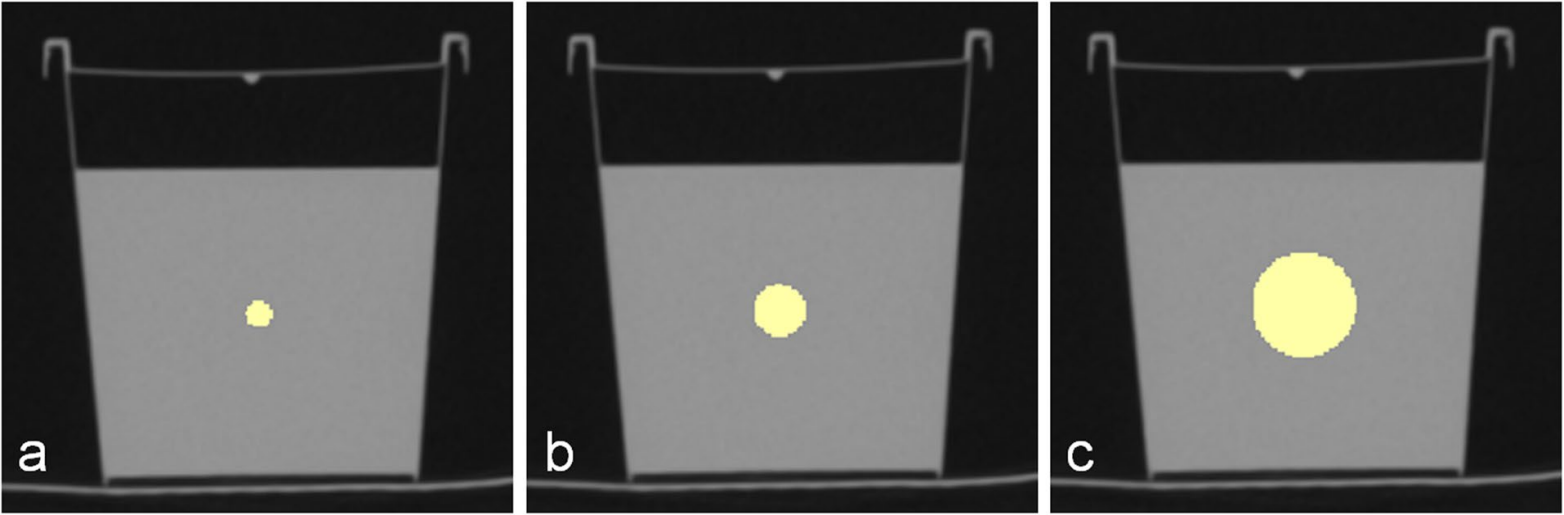
Computed tomography images of the phantom and volume-of-interest (VOI) placement. Differently sized VOIs are drawn into the centre of the phantom. **a** shows a VOI with 4 mm in diameter, **b** with 8 mm, and **c** with 16 mm

Spherical VOIs were drawn into the centre of the phantom of all ten scans using 3D Slicer (3D Slicer, Version 4.10.0, http://www.slicer.org), as shown in Fig. 1. VOI diameters were set to 4, 8, and 16 mm because their size should be double and four times the voxel size of the parametric maps. A voxel size of 4 mm^3^, on the other hand, was chosen because the largest VOI should still be safely placeable centrally in the phantom, limiting the maximum VOI size to 16 mm in diameter. All features available in PyRadiomics (Version 3.0.1) [27] except for the shape features were extracted (settings as suggested by the developers [24], with binWidth 25, voxelArrayShift 1000, and correctMask true). We excluded shape features from our analysis, as their behaviour at different VOI sizes is obvious. A total of 93 features were extracted, 18 first-order features (energy, total energy, entropy, minimum, maximum, mean, median, interquartile range, range, mean absolute deviation, robust mean absolute deviation, root mean squared, skewness, kurtosis, variance, uniformity, 10th percentile, and 90th percentile). The second- and higher-order feature classes comprised the following: 24 grey level co-occurrence matrix (GLCM), 14 grey level dependence matrix (GLDM), 16 grey-level run-length matrix (GLRLM), 16 grey level size zone matrix (GLSZM), and 5 neighbouring grey tone difference matrix (NGTDM).

### Calculating the parametric maps

Because the calculation of the parametric maps requires considerable computing power, the tool by Kim et al. [25] was adapted to run on Google Colaboratory (https://colab.research.google.com). This significantly shortened the computation time and enabled execution in the background. This step, however, went against the initially intended concept of ease of use, although it offered the aforementioned advantages for the current study. The voxel size was set to 4 mm to match the smallest VOI that was considered for the feature extraction. The script for Google Colaboratory can be found in the supplementary material (textfile S1).

### Feature retrieval from the parametric maps

**Fig. 2:**
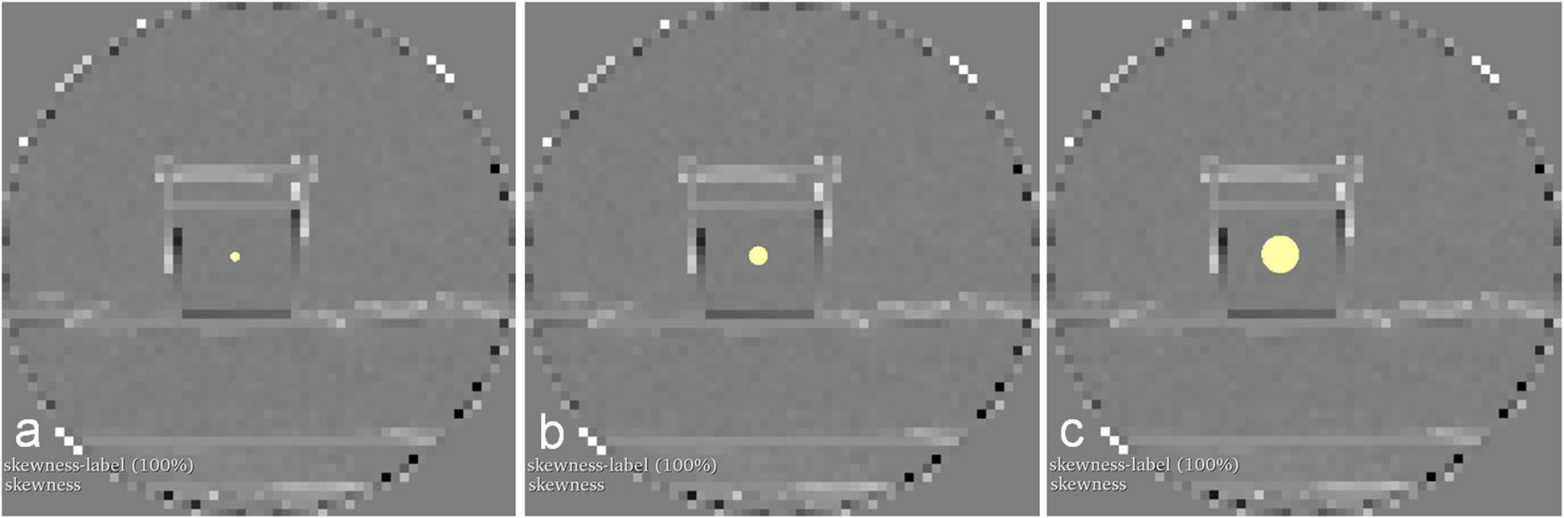
Extraction of feature quantity from parametric maps. Slices of the parametric maps of the first-order feature skewness are shown. We copied volumes of interest (VOIs) with 4 (**a**), 8 (**b**), and 16 (**c**) mm diameter from the conventional feature extraction onto the maps. Feature quantities, visualised by the map, were extracted with PyRadiomics using the mean value of the VOIs

Maps were computed for every feature. The differently sized VOIs used for the conventional extraction were copied onto the maps to maintain their position, as shown in Fig. 2. PyRadiomics was then again used to retrieve the feature value by only considering the mean.

### Statistical analysis

Statistical tests were performed using R (version 3.5.1) [28]. We performed a univariate analysis with a pairwise Mann-Whitney *U*-test with Bonferroni correction to assess differences between the varying VOI sizes (4 and 8 mm, 4 and 16 mm, and 8 and 16 mm VOIs). A *p*-value < 0.05 was considered for statistical significance. The overall concordance correlation coefficients (OCCC), according to Lin et al. and Barnhart et al. [29–31], were calculated to assess the multivariable agreement between various variables of interest using the epiR package for R. We considered features with an OCCC ≥ 0.85 as stable, as this cutoff had been proposed in a recent study regarding feature reproducibility [18]. We calculated OCCCs once to assess agreement among the VOI sizes 4, 8, and 16 mm (OCCCs_4–16_) and once for the VOI sizes 8 and 16 mm (OCCCs_8,16_). Statistical testing was applied to the results of the conventional feature extraction and the results of the parametric maps.

## Results

### Conventional features

Conventionally extracted, 55 features showed significant differences between the VOI sizes, thereof 8 first-order features (*p* ≤ 0.04). All OCCCs showed poor agreement (< 0.85). Detailed results are listed in the supplementary material (Table S2).

### Parametric maps

**Fig. 3:**
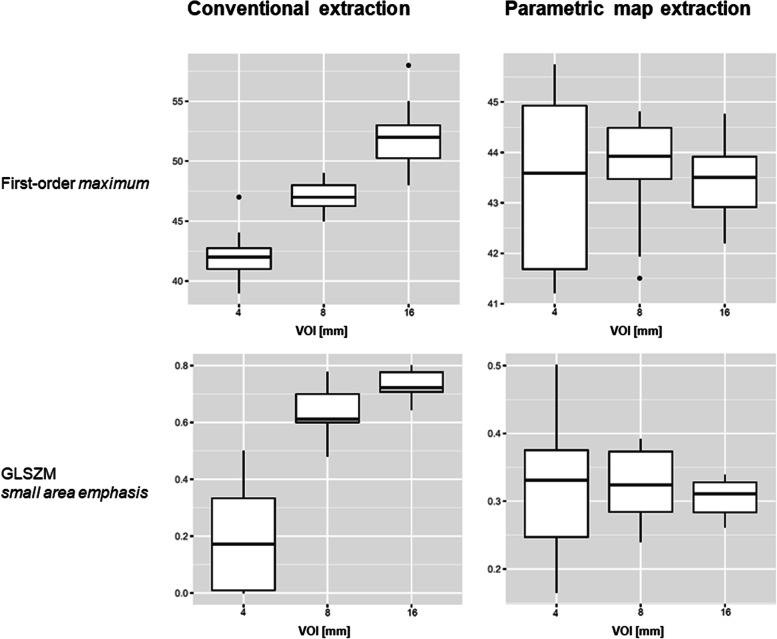
Boxplots of features extracted conventionally and from parametric maps. Exemplary boxplots of the first-order feature maximum and the higher-order feature GLSZM small area emphasis across the different volume-of-interest (VOI) sizes. The left column shows boxplots of the conventional feature extraction with a wide variety of results across the VOI sizes, affecting the median. The same features are shown on the right, but values were retrieved from the parametric maps. Please also note the range on the *y*-axis. Boxplots for all features are provided in the Supplementary material (Fig. S6 contains boxplots of conventionally extracted features and Fig. S7 of extraction from parametric maps)

**Fig. 4:**
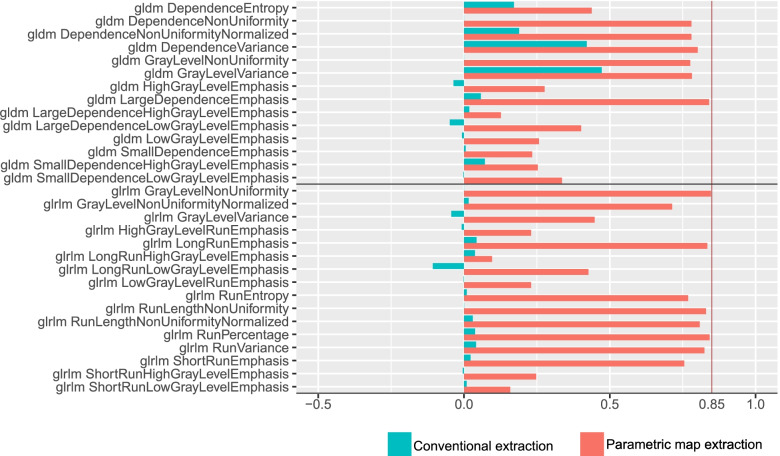
OCCC_8,16_ of higher-order features: conventional and parametric map extraction. Barplots of GLDM and GLRLM features are shown. The green bars illustrate the OCCC_8,16_ values of the conventional feature extraction. The red bars depict the OCCC_8,16_ values when extracted from the parametric maps. The red line at 0.85 indicates excellent agreement. The OCCCs from parametric maps show a substantial increase in stability across the 8 and 16 mm VOI diameters (Supplementary Fig. S8 contains barplots of the OCCC_4–16_ values of all features and Fig. S9 of the OCCC_8,16_ values of all features). *OCCC*, Overall concordance correlation coefficient; *OCCC*_*4–16*_, OCCC among the 4-, 8-, and 16-mm sizes of volumes of interest; *OCCC*_*8,16*_, OCCC between the 8- and 16-mm sizes of volumes of interest

None of the features showed significant differences when we compared results for VOI diameters of 4 and 8 mm. For VOI diameters of 8 and 16 mm, we observed significant differences for 8 features (first-order 10th percentile, first-order minimum, first-order variance, GLDM large dependence high grey level emphasis, GLDM large dependence low grey level emphasis, long-run low grey level emphasis, GLSZM small area low grey level emphasis, NGTDM busyness). For VOI diameters of 4 and 16 mm, a significant difference was observed for only one feature (first-order 10th percentile). Figure 3 shows boxplots for the features first-order maximum and glszm small area emphasis illustrating the decrease in significant differences for these features when extracted from the parametric maps. The OCCC of 88 features across VOI sizes of 4, 8, and 16 mm and of 89 features across 8 and 16 mm increased when we compared parametric maps with conventional features. Furthermore, the OCCC_8,16_ showed an excellent agreement for three features (first-order 90th percentile, GLCM cluster shade, GLRLM nonuniformity). Figure 4 shows the increasing agreement of the OCCCs of gldm and glrlm features when features were extracted from parametric maps. The results of statistical comparisons are provided in supplementary material S3. An overview of OCCC values for conventional features and parametric maps is given in supplementary materials S4 and S5 (Table S4 for values of OCCC_4–16_ and S5 for values of OCCC_8,16_).

## Discussion

The results of the present study show that converting CT images into parametric maps before extracting radiomic features almost resolves significant differences caused by different VOI sizes. In addition, there is a substantial increase in the stability across VOI sizes, as indicated by the improvement of the OCCC values.

When extracted from the original CT data, many features showed significant differences between the three VOI sizes, although they were derived from the same texture. Transferred to a radiomic study, this could simulate a false correlation with a clinical endpoint only by including differently sized VOIs, increasing the demand for a control tool for the VOI confounding effect. Considering our findings for VOIs from 4 and 8 mm, such false results could be avoided if parametric maps were used.

The software tool by Kim et al. [25] that we applied dissembles an image into voxels of a fixed size. The feature is then calculated for each voxel, and the brightness of the voxel in the map reflects the feature quantity at the same position as in the original image [25]. Hence, we can quickly and directly retrieve the feature quantity from the map by drawing a region of interest or VOI. As features are calculated for voxels of the same size, any effects due to different VOI sizes are eliminated. This may not only affect obvious volume confounding but may also reduce the impact of other disturbing factors, such as artifacts, which can alter the results by producing outliers. When directly extracted from a radiological image, a single outlier in the segmented volume may already have significant impact. Applying the parametric maps, outliers then only affect individual voxels and no longer the entire VOI. For example, GLCM and GLRLM features are prone to outliers [32], and these feature classes showed a considerable increase in reproducibility when derived from the parametric maps (for the GLRLM features, see Figs. 3 and 4).

In this context, it is also interesting that, in particular, the number of significantly different features between the VOI sizes of 4 and 8 mm was reduced. Since the voxel size was set to 4 mm^3^, it is conceivable that the reduction of confounding factors for VOIs of twice the voxel size (*i.e.,* up to 8 mm) has a greater effect than for VOIs of four times the voxel size. If we consider a 4-mm VOI placed exactly in the centre of a 4-mm voxel, quantities are only defined by this voxel. And if an 8-mm VOI is placed in the centre of the same voxel, approximately 24% of its volume is already defined by the same voxel (*i.e.,* the cubic voxel of 4-mm edge length within the spherical VOI of 8 mm in diameter). Considering the 16-mm VOI, however, this share amounts for only approximately 3%.

Other groups have reported comparable results regarding the normalisation of voxel size before feature calculation. Shafiq-ul-Hassan et al. [33, 34] improved the stability of radiomic features by normalisation of voxel size of the underlying image. Among other methods, also Larue et al. [35] and Ligero et al. [36] attempted to increase feature robustness by resampling to isometric voxels. Our approach, however, is different. We calculate features for voxels of a fixed size without altering the original image data beforehand to produce parametric maps, and feature values are later retrieved from these maps. The approaches by Shafiq-ul-Hassan et al. [33, 34], Larue et al. [35], and Ligero et al. [36] normalise or resample the pixels/voxels of the original image, and still, the segmented volume is considered as a whole for the feature calculation.

Another viable approach was presented by Lu et al. [37], who aimed to establish a CT radiomics signature of renal clear cell carcinoma and detected radiomic features impacted by tumour size. They suggested a stepwise correction for features susceptible to different tumour volumes, excluding 473 of the initially included 1,160 features. The stepwise elimination of nonreproducible features as a plausible concept was also applied in other studies [3, 38]. Still, a radiomics signature across different studies is not applicable if decisive features in one cohort are not reproducible in another setting. Roy et al. [39] investigated a significant impact of tumour volume on radiomic features in breast cancer lesions on magnetic resonance imaging. They attempted to correct for volume dependency by investigating the correlation of the feature with the volume. If the correlation was linear, they normalised the feature by dividing it by tumour volume and by multiplying it, if the feature was inversely proportional. Regarding the nonlinear correlated features, a principal component analysis aiming to identify a radiomic signature that is volume independent was performed. Following dimension reduction, some features still correlated to tumour volume [39]. Hence, volume dependency in the design of studies with radiomic analysis as an endpoint has to be emphasised when including tumours with different volumes [39].

Although the presented approach to eliminate volume dependency shows promising results, the following limitations have to be considered. It is time-consuming to translate a complete CT volume into maps for every single feature. In clinical routine, calculating maps for all features, *e.g.,* for a whole-body scan, would require a considerable amount of computing power. As a reasonable solution, only maps of those features enhancing a specific diagnosis could be calculated by implementing pre-existing study results. However, if sufficient computing power for calculation of all features maps was available, a quick assessment of the feature quantity for other lesions or structures in the image becomes feasible, as the direct readout does not require further software steps. Furthermore, the extraction from the parametric maps improved the concordance, as shown by OCCC values. Yet, only three features actually yielded an excellent OCCC_8,16_. An increase in OCCC values of all features to at least 0.85 would have been desirable. Still, using the maps could safely ban significant differences between the differently sized VOIs for all but 8 features.

Another aspect of the parametric maps compared to conventional extraction is that different anatomical structures can be contained in one voxel. This is one of the reasons why the results from the maps and a conventional extraction will not be the same, although some features show concordant results [25]. For clinical use, this would have to be taken into consideration when selecting the voxel size and should be evaluated in further studies.

**Fig. 5:**
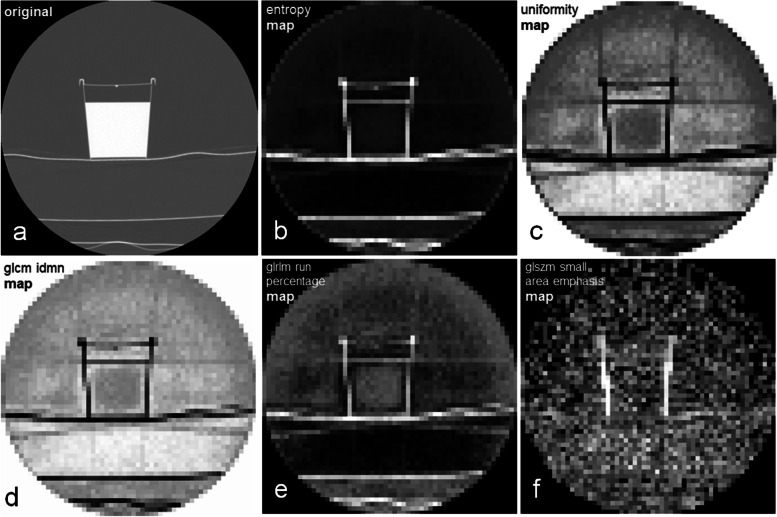
Exemplary parametric maps. The original computed tomography scan of the phantom is shown in the left upper corner (**a**). The exemplary maps represent the quantity of the respective feature. The higher the feature quantity for a voxel, the brighter the voxel appears on the map. The map of GLSZM small area emphasis (**f**) illustrates that the feature quantity of the background is almost similar to the body of the phantom. In the maps of entropy (**b**), uniformity (**c**), GLCM IDMN (**d**), and GLRLM run percentage (**e**), lines appear in the extension of edges and interfaces of the phantom. A high signal in the background can also be observed

Finally, even though it is a by-product of this study, we noted that visualisation of parametric maps seems to help better understand the behaviour of textural features. For example, in some of the maps shown in Fig. 5, the lines in extension of interfaces and edges propagate beyond the phantom through the entire image. Corresponding effects can be expected in a clinical CT examination, but with innumerable interfaces and edges. To correct radiomic features for such complex effects seems extremely difficult. However, as already discussed above, maps with a fixed voxel size may also reduce the impact of other confounding factors besides volume.

In conclusion, converting CT images into parametric maps before extracting radiomic features increases reproducibility across VOI sizes. Furthermore, parametric maps can prevent incorrect significant results attributable to varying VOI sizes. The maps could furthermore visually elucidate complex phenomena of the features throughout the entire image.

## Supplementary Information

**Additional file 1: Textfile S1.** Script for Google Colab. **Table S2.** Results of the Mann-Whitney *U* test for the conventional feature extraction. **Table S3.** Results of the Mann-Whitney *U* test for the parametric map extraction. **Table S4.** Comparison of OCCC_4-16_ values: conventional extraction and parametric maps. **Table S5.** Comparison of OCCC_8,16_ values: conventional extraction and parametric maps. **Fig. S6.** Boxplots of the conventional extraction for all features. **Fig. S7.** Boxplots of the parametric map extraction for all features. **Fig. S8.** OCCC_4-16_ barplots comparing conventional and parametric map extraction. **Fig. S9.** OCCC_8,16_ barplots comparing conventional and parametric map extraction.

## Data Availability

The datasets used and/or analysed during the current study are available from the corresponding author on reasonable request.

## Abbreviations

CT: Computed tomography
GLCM: Grey level co-occurrence matrix
GLDM: Grey level dependence matrix
GLRLM: Grey level run-length matrix
GLSZM: Grey level size zone matrix
NGTDM: Neighbouring grey tone difference matrix
OCCC: Overall concordance correlation coefficient
OCCC_4–16_: OCCC among the 4-, 8-, and 16-mm VOI sizes
OCCC_8,16_: OCCC between the 8- and 16-mm VOI sizes
VOI: Volume of interest
